# Metformin-Induced Mitochondrial Complex I Inhibition: Facts, Uncertainties, and Consequences

**DOI:** 10.3389/fendo.2018.00753

**Published:** 2018-12-17

**Authors:** Eric Fontaine

**Affiliations:** INSERM, LBFA Université Grenoble Alpes, Grenoble, France

**Keywords:** metformin, mitochondria, Complex I, pharmacokinetic, cell death, cancer, permeability transition

## Abstract

Metformin is the most widely prescribed drug to treat patients with type II diabetes, for whom retrospective studies suggest that metformin may have anticancer properties. However, in experiments performed with isolated cells, authors have reported both pro- and anti-apoptotic effects of metformin. The exact molecular mechanism of action of metformin remains partly unknown despite its use for over 60 years and more than 17,000 articles in PubMed. Among the various widely recognized or recently proposed targets, it has been reported consistently that metformin is capable of inhibiting mitochondrial respiratory chain Complex I. Since most of the effects of metformin have been replicated by other inhibitors of Complex I, it has been suggested that the mechanism of action of metformin involved the inhibition of Complex I. However, compared to conventional Complex I inhibitors, the metformin-induced inhibition of Complex I has unique characteristics. Among these, the most original one is that the concentrations of metformin required to inhibit Complex I are lower in intact cells than in isolated mitochondria. Experiments with isolated mitochondria or Complex I were generally performed using millimolar concentrations of metformin, while plasma levels remain in the micromolar range in both human and animal studies, highlighting that metformin concentration is an important issue. In order to explain the effects in animals based on observations in cells and mitochondria, some authors proposed a direct effect of the drug on Complex I involving an accumulation of metformin inside the mitochondria while others proposed an indirect effect (the drug no longer having to diffuse into the mitochondria). This brief review attempts to: gather arguments for and against each hypothesis concerning the mechanism by which metformin inhibits Complex I and to highlight remaining questions about the toxicity mechanism of metformin for certain cancer cells.

## Introduction

Metformin is a drug with pleiotropic effects. It takes part in glucose homeostasis, mainly by inhibiting liver glucose production (1). It also modifies the production of reactive oxygen species and affects cell death processes (2, 3). Most of these effects have been traced to the inhibition of mitochondrial respiratory chain Complex I for two main reasons: First, over the past 20 years, different laboratories have reproducibly observed that metformin inhibits mitochondrial respiratory chain Complex I (4–20). Second, these pleiotropic effects have been reproduced by well identified Complex I inhibitors [gluconeogenesis (21, 22), cell death (18, 23–28)].

However, the mechanism by which metformin affects the activity of Complex I remains debated. In order to clarify whether the different conclusions found in the literature may be due to methodological differences, this review compares results obtained *in vivo* or with intact cells, to results obtained with isolated mitochondria or isolated Complex I. In this last case, authors tend to assume that metformin accumulates in mitochondria, here we will discuss evidence supporting or not this assumption. Finally, since pro- and anti-apoptotic effects of metformin are observed in intact cells, we will examine the role of metformin concentrations as a potential cause of these conflicting observations.

## Metformin Pharmacokinetics

Metformin is a hydrophilic compound charged positively at physiological pH. Its hydrophilicity limits its permeability through lipid membranes. Metformin enters and leaves cells by the presence of several transporters including Organic Cation Transporters (OCTs) and multidrug and toxin extrusion (MATE) transporters (29). This leads to a steady-state concentration of metformin inside cells, depending on both the amount and activity of such transporters as well as metformin plasma concentration.

The pharmacological inhibition or the genetic ablation of OCTs reduce the distribution of metformin to the liver, small intestine and kidney (30–32) while the overexpression of OCT1 in HEK293 and CHO cells increases metformin uptake (30, 33). The pharmacological inhibition or the genetic ablation of MATE1 cause hepatic and kidney accumulation of metformin (32, 34). In humans, the genomic variations of metformin transporters can affect its pharmacokinetics (concentration, clearance, volume of distribution) (35, 36) suggesting that such genomic variations affect metformin concentration in tissues.

Whether the activities of the metformin transporters (i.e., the metformin concentration in tissues) affect the metabolic effects of metformin is not systematically reported in the literature. On the one hand, metformin failed to reduce fasting plasma glucose concentration in OCT1-knockout mice submitted to a high-fat diet for 8 weeks and failed to suppress glucagon-stimulated glucose production in OCT1^−^/^−^ hepatocytes (30). On the other hand, the effect of metformin on glucose tolerance tests was similar in animal controls and OCT1/2-knockout animals (31). A broad variation in clinical efficacy of metformin has long been recognized as well as a reduced function polymorphism of OCT1 in humans. However, if some authors reported a decreased effect of metformin in type-2 diabetes patients carrying reduced function polymorphism of OCT1 (30, 36), others did not observe such a correlation (37, 38).

To the best of my knowledge, no study correlating metformin concentration in tissue (or cells) and metformin-induced Complex I inhibition was ever published.

Drugs that are extensively sequestered in organelles have a very large apparent volume of distribution and a prolonged half-life *in vivo* (39). Metformin is not metabolized and is secreted by the kidneys with a half-life of 1.74–7.3 h in humans depending on the studies (35, 40–42). With a volume of distribution of 1.12 ± 0.08 L/kg in healthy volunteers (40), metformin is not supposed to accumulate dramatically in tissues. The amount of metformin in the liver ranges from 2 to 5 times that of plasma -depending on the studies (32, 35, 42, 43)- and increases up to 10 times that of plasma in small intestinal walls (32).

Thus, the pharmacokinetic studies indicate that metformin enters but does not accumulate in large amounts in cells. Whether its metabolic activity depends on its diffusion inside the cells is supported by several but not all studies.

Once in the cell, as metformin inhibits Complex I it is tempting to speculate that metformin penetrates the mitochondria. The composition of the mitochondrial matrix (the space delimited by the inner mitochondrial membrane) is different from that of the cytosol. In order to maintain such a different metabolite composition, the inner membrane is impermeable to almost all hydrophilic molecules which enter or leave the mitochondria through specific transporters. Among the numerous recognized mitochondrial carriers, no specific carrier for metformin has been identified yet.

Despite this, many authors have hypothesized that metformin accumulates in mitochondria (5, 13, 15, 44). This scenario may reconcile the observation that millimolar concentrations of metformin are necessary to inhibit Complex I in isolated mitochondria (see below) while, when used at the therapeutic dose, the plasma metformin concentration remains in the micromolar range in both humans and animals (31, 36, 42).

**Figure 1:**
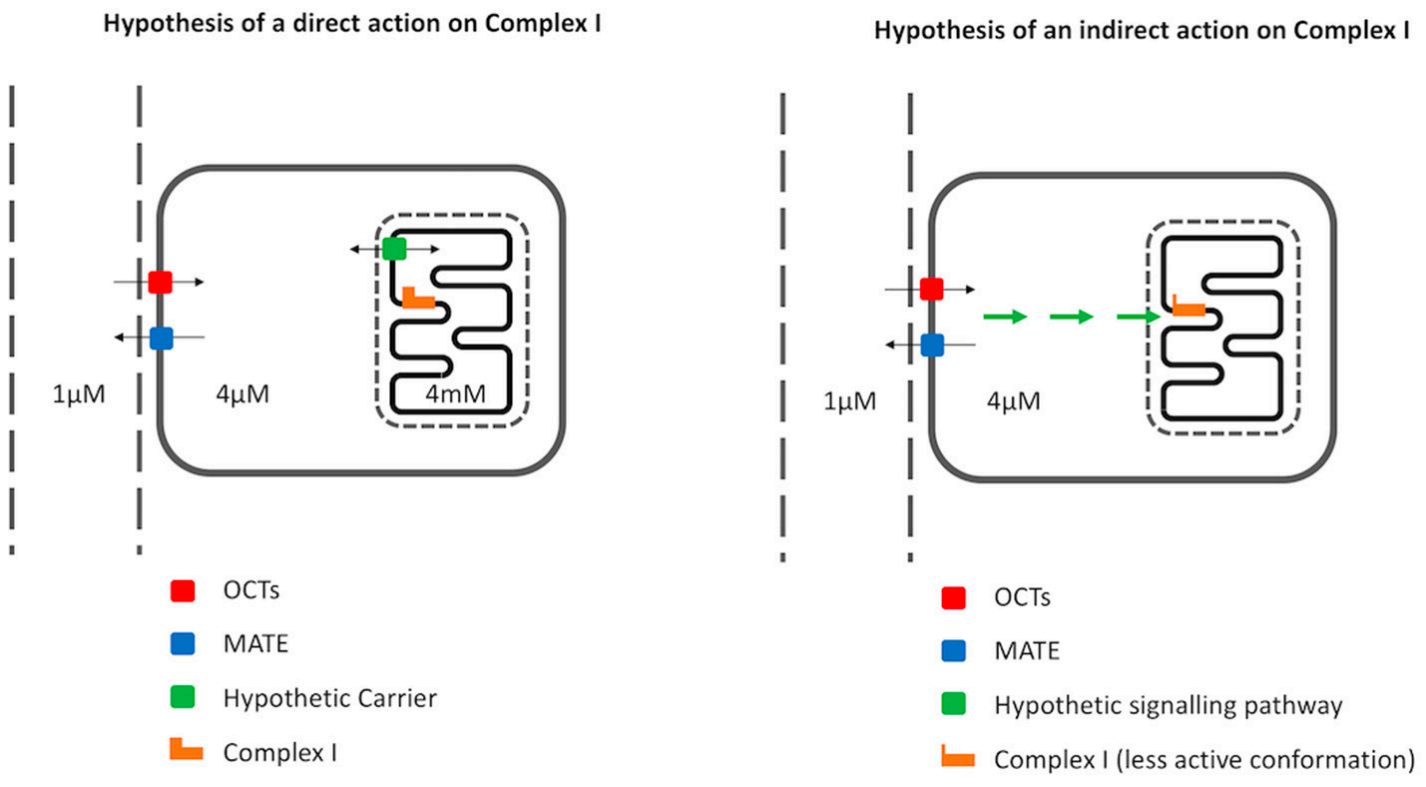
Mechanism of action of metformin on complex I: the direct hypothesis and the indirect hypothesis. Metformin enters cells via Organic Cation Transporters (OCTs) and leaves cells via multidrug and toxin extrusion (MATE) transporters. Assuming a plasma membrane potential of 35 mV and a passive mechanism of OCTs and MATE, the cytosolic metformin concentration is expected to be 4 times that of the plasma concentration. In the direct hypothesis **(left)**, metformin is supposed to enter mitochondria via a hypothetical carrier reaching a matrix concentration 1,000 times that of the cytosol for mitochondrial membrane potential of 180 mV. In the indirect hypothesis **(right)**, metformin does not enter mitochondria but stimulates a hypothetical signaling pathway that eventually modifies Complex I conformation, making it less active.

From a theoretical point of view, this hypothesis is plausible. Indeed, because the mitochondrial respiratory chain transfers protons from the matrix to the intermembrane space, mitochondria build up and maintain an electrical mitochondrial membrane potential that drives the accumulation of positively charged molecules into mitochondria, provided the molecule crosses the membrane. In these conditions, Nernst equation indicates that for a physiological mitochondrial membrane potential of −180 mV the thermodynamic equilibrium is reached after a 1,000-fold accumulation of a positively charged molecule if the molecule has one charge. Since metformin is a positively charged molecule and assuming the presence of a still unknown carrier for metformin in the inner membrane, its mitochondrial concentration could reach the millimolar range despite a cytosolic concentration within the micromolar range (see Figure 1). In addition, assuming a plasma membrane potential of −36 mV and the absence of kinetic constraints on metformin transporters (OCT and MATE), the cytosolic concentration of metformin would be 4 times that of plasma.

However, the hypothesis that metformin accumulates in mitochondria contradicts several observations.

First of all, the accumulation of numerous positive charges in the matrix compensated by proton extrusion by the respiratory chain, should lead to a collapse of mitochondrial membrane potential associated with an increase in delta pH. However, note that metformin did not depolarize isolated mitochondria (8).

Secondly, assuming that the total mitochondrial volume represents approximately 20% of hepatocytes, a 1,000-fold accumulation of metformin inside mitochondria would represent an approximately 200-fold accumulation of metformin in liver (without accounting for accumulation in the cytosol). Such an accumulation is 2 orders of magnitude higher than that measured by several groups (32, 35, 42, 43).

Thirdly, a large mitochondrial accumulation is not compatible with the low volume of distribution of metformin and its short half-live (see above).

Fourthly, using radioactive [^14^C] metformin, the radioactivity was not found to accumulate in liver mitochondria of rats treated orally with metformin (45) and no radioactivity was measured inside mitochondria when *Xenopus laevis* oocytes were exposed to concentrations of metformin that led to Complex I inhibition (6). Importantly, Complex I remained inhibited after mitochondrial isolation. Although this result does not definitively exclude a possible accumulation of metformin in mitochondria as a cause of Complex I inhibition (accumulated metformin may diffuse during the isolation procedure), it rules out the hypothesis that the inhibition of Complex I by metformin requires metformin inside mitochondria.

In summary, unlike the less hydrophilic biguanides (46), the accumulation of metformin inside the mitochondria is not supported by direct measurements, is not consistent with the pharmacokinetic data, and would require a transporter that has not yet been discovered.

Derivatives combining a molecule of metformin at different alkyl chain lengths containing a triphenylphosphonium cation (a liposoluble cation known to accumulate in mitochondria according to membrane potential) have been synthesized (47, 48) in order to increase the anti-cancer effect of metformin (see below). These different compounds accumulate in cells (47), depolarize mitochondria (48) and inhibit Complex I with an IC_50_ in the micromolar range (47, 48), which according to Nernst equation is consistent with the accumulation of compounds in the mitochondrial matrix at a concentration in the millimolar range. If metformin accumulated spontaneously in the mitochondria, the addition of molecules targeting the mitochondria would be unnecessary, which is clearly not the case.

## Characteristics of Complex I Inhibition According to the Models Used

Although this may seem odd, it has been reported by several different laboratories that the concentrations required to inhibit Complex I are lower for intact cells than for isolated mitochondria (4–6, 13, 19). Note however that the characteristics of Complex I inhibition reveals some differences depending on whether metformin acts on intact cells (animal models, infused organs, isolated cells) or directly on isolated mitochondria or isolated Complex I (see Table 1).

**Table 1:** Main differences in the characteristics of Complex I inhibition according to the model used.

	**Live animal, perfused organ, intact cells**		**Isolated mitochondria, isolated Complex I**	
		**References**		**References**
Speed of inhibition	Time dependent	(4) (5)	Immediate (minutes)	(14) (13)
Type of inhibition	Partial	(4) (5) (18)	Total	(13)
Affinity	Apparent IC_50_ approximately 1 mM after 30 min in isolated rat hepatocytes Apparent IC_50_ 250 μM and 330 μM after 6 h incubation in 143B and HepG2 cells respectively	(4) (13)	Apparent IC_50_ 15 mM in isolated mitochondria IC_50_ 79 mM in sub-mitochondrial particles IC_50_ 66 mM in immunocaptured Complex I IC_50_ 19 mM in isolated Complex I	(5) (5) (9) (13)
Inhibition in State-3?	Yes	(5) (11) (12) (14) (15)	Yes	(5) (8) (9) (10) (12) (14) (15)
Inhibition in State-4?	Yes	(4) (7) (14)	No	(4) (8) (10) (15)
Inhibition after uncoupling	Yes	(4) (7) (11) (14) (19)	No	(4) (15) (19)
Inhibition after the removal of metformin	Yes	(4) (14) (20)	No for metformin concentration ≤ 2 mM	(13)
NADH/NAD^+^	Increases	(4) (5) (18)	Decreases	(19)

The incubation of isolated Complex I or submitochondrial particles in the presence of millimolar concentrations of metformin leads to an inhibition of Complex I that can be complete (13) with an IC_50_ ranging from 19 to 79 mM depending on laboratories (5, 9, 13). It should be noted that there is no membrane potential in these particular conditions of incubation, thus no possibility of metformin accumulation. In other words, the concentrations tested are the actual concentrations to which Complex I is exposed.

The incubation of isolated mitochondria in the presence of millimolar concentrations of metformin leads to a rather fast (within a few minutes) inhibition of Complex I with an “apparent” IC_50_ also in the millimolar range (5).

This observation is not easily reconcilable with the proposal that metformin accumulates in mitochondria. Indeed, assuming that metformin did accumulate in mitochondria, Complex I inhibition would have been observed at micromolar concentrations of metformin (corresponding to millimolar concentrations inside mitochondria), which has not been reported. One could argue that at millimolar concentrations of metformin, the inhibition of Complex I would depolarize mitochondria, preventing metformin accumulation. However, it has been shown that millimolar concentrations of metformin did not depolarize isolated mitochondria (8).

In these particular conditions of incubation (isolated mitochondria exposed to millimolar concentrations of metformin), it has to be noted that the inhibition of Complex I is observed almost exclusively during ATP synthesis (also called State 3) and disappears when mitochondria are depolarized (uncoupled State) or at rest (also called State 4) (4, 8, 15). Such behavior is not observed with rotenone (the reference inhibitor of Complex I) but is typical of biguanide-induced inhibition of Complex I in isolated mitochondria (49).

It has been proposed that the reason why the inhibition is not observed after uncoupling might be due to the fact that the driving force for metformin accumulation within mitochondria disappears in these particular conditions. Although, as stated above, the accumulation of metformin in mitochondria is not supported by any evidence, this hypothesis does not explain the lack of inhibition in State 4, a situation in which the driving force (the membrane potential) is higher than in State 3.

Curiously, it has been reported that the inhibition of oxygen consumption in isolated mitochondria is accompanied by NADH oxidation (19). This observation is not expected in case of a simple Complex I inhibition, suggesting an uncoupling effect of metformin in this particular condition.

The incubation of intact cells in the presence of metformin leads to a slower inhibition of Complex I depending on metformin concentration (hours are required for micromolar concentrations of metformin) (5, 50). Contrary to what is observed in isolated Complex I, the inhibition is not total and plateaus at approximately 40% of the Vmax (4). Consistent with a pure effect on Complex I, the inhibition leads to an increase in the NADH/NAD^+^ ratio (as assessed by the Lactate/pyruvate and 3-hydroxybutyrate/ acetoacetate ratios) (4, 5). Importantly, once cells are permeabilized (i.e., once mitochondria can be studied as if they were isolated) the inhibition is observed in State 3, but also in State 4 and after uncoupling (4, 11, 19). Finally, Complex I remains inhibited in mitochondria isolated from either rat exposed to metformin or liver perfused with metformin, even after uncoupling (4, 14) or when NADH:quinone oxidoreductase activity (i.e., Complex I activity) is studied directly using broken mitochondria (4). Note that the isolation procedure removes most of (if not all) the free metformin, while uncoupling (either chemical or after inner membrane rupture) would release the putative accumulated metformin. Although these results do not exclude a possible binding of metformin in mitochondrial membrane, they rule out the hypothesis that the inhibition of Complex I by metformin could depend on membrane potential.

## Other Mitochondrial Effects Of Metformin

In intact cells the inhibition of oxygen consumption is strictly located on Complex I. This conclusion comes from the observation that metformin has no effect on oxygen consumption when electrons feed the respiratory chain downstream Complex I (using succinate for example) regardless of the respiratory State (3, 4 and uncoupled) (4).

On the contrary, using isolated mitochondria and millimolar concentrations of metformin, some authors reported inhibitory effects on complexes III and IV (16). High concentrations of metformin have been reported to inhibit ATP hydrolysis but not ATP synthesis (13), suggesting an unconventional effect on the ATP synthase.

Some evidence suggests that Complex I can interact with ATP synthase (51). So we may infer that in this particular condition of incubation (isolated mitochondria exposed to millimolar concentrations of metformin), ATP synthesis possibly sensitizes Complex I to metformin. Although speculative, this personal suggestion could account for the observation that millimolar concentrations of metformin inhibit Complex I almost exclusively in State 3.

In summary, on intact cells metformin acts slowly but the effect is visible at micromolar concentrations. The inhibition affects only Complex I in all the respiratory states and does not depend on mitochondrial membrane potential. On isolated mitochondria (or isolated Complex I), metformin acts rapidly but the effect requires millimolar concentrations. The inhibition does not only affect Complex I and Complex I inhibition is not observed in all the respiratory states.

## Where Does Metformin act on Complex I?

The respiratory chain is a sequence of redox reactions which couple an electron flux with a vectorial transfer of protons. Mammalian respiratory chain complex I is a large protein complex with at least 45 subunits. It includes a hydrophobic part embedded in the inner membrane involved in proton transfer and a hydrophilic part protruding into the matrix in which electrons pass from NADH to ubiquinone via a succession of redox reactions. Complex I inhibitors rotenone and piericidin bind at, or close to, the ubiquinone binding site, inhibiting both electron flux and proton extrusion. Using artificial electron acceptors, a rotenone-insensitive NADH oxidation which is not coupled with proton pumping (i.e., a non-physiological pathway) can occur in Complex I.

Using isolated Complex I and millimolar concentrations of metformin, it has been shown that metformin does not inhibit NADH oxidation due to artificial electron acceptors, behaves as a non-competitive inhibitor of the physiological electron pathway and preferentially binds Complex I when the enzyme is in its “deactive” conformation (13). However, the exact localization where metformin acts in this condition of incubation remains unknown. Moreover, the exact mechanism leading to the inhibition of Complex I in intact cells using micromolar concentrations of metformin and where exactly it inhibits the electron flux in Complex I has not been reported.

## Hypothetical Mechanisms of Action

To account for the fact that the concentration of metformin required to observe the inhibition of Complex I on whole cells is lower than the concentration required to observe the inhibition on mitochondria, two hypotheses have been proposed in the literature (see Figure 1).

The first one (in chronological order, but second in popularity) proposes that *in vivo* and in intact cells, metformin triggers a signaling pathway that in turn induces the inhibition of Complex I (4). Although such a signaling pathway is yet unknown, it has been reported that Complex I exists in two different functional conformations (active and inactive) (52), while reactive thiols of several Complex I subunits have been identified as targets for post-translational modifications (53, 54). However, whether metformin affects reactive thiols in Complex I has not been published yet.

The second hypothesis necessarily involves an accumulation of metformin in the mitochondria that would be driven by mitochondrial membrane potential. Although proposed by several authors, this hypothesis is not yet supported by any evidence (see above).

## Effects of Metformin-Induced Complex I Inhibition on Cell Death Processes

Apparently contradictory effects are found in the literature regarding the effects of metformin on cell death. Some authors have put forward its protective effects against cell death (3) while others have reported its induction of cell death especially in cancer cells (2). Yet, all of them have concluded that the observed effects are due to the inhibition of Complex I (see below).

## Metformin Prevents Cell Death When it is due to PTP Opening

The permeability transition pore (PTP) is a channel located in the inner membrane normally closed in order to maintain a high mitochondrial membrane potential required for ATP synthesis. Once permanently opened, the membrane potential collapses (55), leading to a drastic inhibition of ATP synthesis. Beyond this uncoupling effect, PTP opening has many other effects: It allows the thermodynamic equilibrium of the mitochondrial and cytosolic redox potentials, leading to an increase in cytosolic NAD(P)H concentration (56). It partly inhibits Complex I (57), reallocating the electron flux for the production of reactive oxygen species (58). Finally, it leads to the release of mitochondrial pro-apoptotic proteins both in isolated mitochondria (secondary to mitochondrial swelling leading to the rupture of the outer membrane) (59) and in intact cells (most probably by a distinct but still unknown mechanism) (56, 60–62).

As there are several signaling pathways involved in cell death, there are many factors activating these pathways. To discriminate whether a given condition leading to cell death involves PTP opening or not, experiments are performed in the presence or absence of a recognized PTP inhibitor (generally cyclosporine A, but not exclusively). Using this approach, it has been reproducibly observed that PTP opening occurs when cell death is triggered by calcium overload or oxidative stress (63).

The molecular nature of the PTP has long been a subject of dispute but recent and compelling data from different laboratories suggest that the PTP might involve ATP synthase (51, 64). Surprisingly, the reference Complex I inhibitor rotenone has been shown to inhibit PTP opening in all the tested cells and tissues (either spontaneously or in the presence of cyclosporine A) (23, 65). Although rotenone induces an energetic stress, it also prevents cell death in the same models as cyclosporine A (23) and does inhibit Complex I and PTP opening with a similar concentration dependence (65). Piericidin, another well recognized Complex I inhibitor also inhibits PTP opening (23). Thus, the activity of Complex I can be said to be a regulator of PTP opening. Moreover, several ubiquinone analogs (known to bind with Complex I among others) have been proved to regulate PTP opening and cell death (57, 66–69).

Knowing that metformin partly inhibits Complex I, we tested whether it also inhibited PTP opening and related cell death. We found that, metformin was less potent than rotenone but also inhibited PTP opening (50). Suggesting a common mechanism of action with rotenone, the effect of metformin was not additive with that of rotenone, whereas it was additive with that of cyclosporine A (65). At present, metformin has been shown to prevent PTP opening in endothelial cells (50), KB cells (7), INS-1 insulinoma cells (61), HeLa cells (65), LNCaP cells (70), A375 cells (70), primary cortical neurons (71) and kidney mitochondria (72). Accordingly, metformin prevents cell death induced by oxidative stress in endothelial cells (50) and KB cells (7), etoposide in primary neurons (71), gentamicin in kidneys (72), hyperglycemia in endothelial (50) and INS-1 cells (61), hyperfructosemia in INS-1 cells (61) and ischemia reperfusion in INS-1 cells (73). Many other works have found a protective effect of metformin (particularly during oxidative stress or ischemia reperfusion injury) without having studied the role of the PTP (18, 74–77).

## Anti-Neoplastic Effects Of Metformin

Although PTP opening irremediably leads to cell death, PTP opening is not mandatory to kill cells as cells can die with a closed PTP. Although Complex I inhibition prevents PTP opening-related cell death (see above), it can also induce cell death in several models. Indeed, it has been repetitively reported that rotenone (25) or biguanides (including metformin) can induce cell death, especially in cancer cells (15, 17, 20, 78).

Cancer cells are known to be generally highly glycolytic (Warburg effect) and are thus not supposed to be very sensitive to mitochondrial poison. But is it so simple? As soon as cells consume oxygen at the mitochondrial level, they are supposed to produce mitochondrial ATP. Thus, even if the proportion of mitochondrial ATP production is reduced in cancer cells, this mitochondrial ATP production exists and its reduction could be toxic. Supporting this proposal, it has been reported that metformin inhibits the proliferation of HCT116 p53^−/−^ cancer cells in the presence of glucose, while it induces cell death in case of glucose deprivation (15). Moreover, the effect of metformin is totally prevented by the overexpression of a metformin-resistant *Saccharomyces cerevisiae* NADH dehydrogenase NDI1 (15), very elegantly demonstrating that the toxicity of metformin is due to its effect on Complex I.

The suggestion that metformin's toxicity is related to an energetic stress raises several questions: Why is metformin less toxic in non-cancer cells that are yet more dependent on mitochondrial ATP production? How can metformin protect against PTP-induced cell death despite its effect on ATP production? In other words, what triggers that a same inhibition of Complex I either prevents or induces cell death?

Again, part of the answer could be found in the comparison of metformin concentrations. While millimolar concentrations of metformin are generally used to induce cell death *in vitro*, micromolar concentrations are sufficient to prevent PTP-opening induced cell death. Although it has been shown that cellular energy status is inversely correlated with metformin concentrations (11, 79), a 24-h incubation with 100 μM metformin did not affect the AMP/ATP ratio in primary cultured hepatocytes (11). This suggests that the metformin concentration used to prevent PTP opening (100 μM, overnight) was not sufficient to induce a lethal decrease in energy status. On the contrary, this confirms that the concentrations used to kill cells dramatically affect the energy status. Note however that some authors have reported an anti-apoptotic effect even at millimolar concentrations of metformin, suggesting that some cells are able to overcome energy stress (75, 80).

However, if the mechanism by which metformin kills isolated cells can be traced to a collapse in energy status, the concentrations that prevent cancer growth in animal models are in the micromolar range. The practical assumption of metformin accumulation in mitochondria has obviously been retained, but one can wonder: why are normal cells preserved? Alternative or complementary explanations must exist. Among them, it has been proposed that the effect of metformin in animal models is indirect (for example due to a decrease in blood insulin concentration) (2). It is also possible that the accumulation of metformin or the sensitivity of Complex I to metformin is higher in cancer cells than in normal tissues (personal hypothesis). As far as I know, these assumptions have not yet been tested.

## Conclusions and Proposal

As explained several times in this manuscript, the concentration with which experiments were conducted is the main misleading point regarding the effect of metformin on Complex I. On the one hand, it is obvious that the assumption that metformin accumulates in mitochondria suits many authors. This hypothesis can bridge the gap between concentrations measured *in vivo* and those used *in vitro*. On the other hand, two different laboratories that attempted to measure such an accumulation put forward a total absence of metformin accumulation in mitochondria (6, 45) in which Complex I was nevertheless inhibited (6). Furthermore, although the pharmacokinetic data are indirect evidence, they are not compatible with an accumulation of metformin in mitochondria.

Facing the facts, one must admit that there is either a technical mistake in the studies that did not find metformin accumulation in mitochondria or there is absolutely no experiment performed at millimolar concentrations of metformin that reflect what occurs *in vivo*. This includes a lot of articles both on its antidiabetic role and on its anticancer effect. There is an urgent need to solve this problem for good, and this could be performed easily by fast cell fractionation coupled to mass spectrometry (or other technics to detect metformin) in order to confirm if metformin is found in large amount in mitochondria of cells exposed to metformin. Currently, the published evidence does not support the generally accepted hypothesis of metformin accumulation in mitochondria.

## Author Contributions

The author confirms being the sole contributor of this work and has approved it for publication.

### Conflict of Interest Statement

The author declares that the research was conducted in the absence of any commercial or financial relationships that could be construed as a potential conflict of interest.
